# Evaluation of Clinical and Immunological Markers for Predicting Virological Failure in a HIV/AIDS Treatment Cohort in Busia, Kenya

**DOI:** 10.1371/journal.pone.0049834

**Published:** 2012-11-21

**Authors:** Cecilia Ferreyra, Oliver Yun, Nell Eisenberg, Elena Alonso, Ashimosi S. Khamadi, Matilu Mwau, Martha Kihara Mugendi, Ana Alvarez, Elena Velilla, Laurence Flevaud, Mireia Arnedo, David Dalmau, Paul Roddy, Andrea Bernasconi, Pedro Pablo Palma

**Affiliations:** 1 Médecins Sans Frontières, Operational Center Barcelona Athens, Barcelona, Spain; 2 Kenya Medical Research Institute (KEMRI), Nairobi, Kenya; 3 Hospital Clinic de Barcelona, IDIBAPS, Barcelona, Spain; 4 Hospital Universitari Mutua de Terrassa, Terrassa, Spain; German Primate Center, Germany

## Abstract

**Background:**

In resource-limited settings where viral load (VL) monitoring is scarce or unavailable, clinicians must use immunological and clinical criteria to define HIV virological treatment failure. This study examined the performance of World Health Organization (WHO) clinical and immunological failure criteria in predicting virological failure in HIV patients receiving antiretroviral therapy (ART).

**Methods:**

In a HIV/AIDS program in Busia District Hospital, Kenya, a retrospective, cross-sectional cohort analysis was performed in April 2008 for all adult patients (>18 years old) on ART for ≥12 months, treatment-naive at ART start, attending the clinic at least once in last 6 months, and who had given informed consent. Treatment failure was assessed per WHO clinical (disease stage 3 or 4) and immunological (CD4 cell count) criteria, and compared with virological failure (VL >5,000 copies/mL).

**Results:**

Of 926 patients, 123 (13.3%) had clinically defined treatment failure, 53 (5.7%) immunologically defined failure, and 55 (6.0%) virological failure. Sensitivity, specificity, positive predictive value, and negative predictive value of both clinical and immunological criteria (combined) in predicting virological failure were 36.4%, 83.5%, 12.3%, and 95.4%, respectively.

**Conclusions:**

In this analysis, clinical and immunological criteria were found to perform relatively poorly in predicting virological failure of ART. VL monitoring and new algorithms for assessing clinical or immunological treatment failure, as well as improved adherence strategies, are required in ART programs in resource-limited settings.

## Introduction

Substantial progress has been made over the last several years in the number of people receiving antiretroviral therapy (ART) for HIV/AIDS treatment. From a baseline of approximately 400,000 people receiving ART in low- and middle-income countries (LMICs) in December 2003, more than 5 million people were receiving treatment by the end of 2009 [1], [2], [3]. Scale-up in sub-Saharan Africa was most dramatic: from 100,000 people on ART at the end of 2003 to 3.9 million people at the end of 2009 [3]. Despite these extraordinary gains, global coverage of ART in LMICs remains at 36% of the estimated overall need at the end of 2009 [3].

High mortality in the early months of treatment [4] and low rates of retention [5] remain problematic for resource-poor settings. However, immunological, virological, and survival outcomes are encouraging in LMICs [6], [7]. The public health approach promoted by World Health Organization (WHO) allowed the expansion of treatment [8], [9], but led to new challenges, such as early and accurate detection of treatment failure.

In LMICs where routine viral monitoring is limited, clinicians follow WHO recommendations to define treatment failure [8], [9], [10]. Lack of access to viral load (VL) testing in most LMICs has led to dependence on clinical and immunological markers to detect treatment failure, an increasing problem in the era of “switch from D4T to TDF” as recommended by WHO. Concerns surround the “limitations of clinical and immunological monitoring for diagnosing treatment failure” and “premature or unnecessary switching to expensive second-line ART [8].”

In this study we analyzed the performance of WHO criteria for clinical and immunological failure as surrogate measures for virological treatment failure in a context where VL testing is not widely available.

## Methods

### Study Population

In 2003, Médecins Sans Frontières (MSF) began an ART program in Busia District Hospital, Kenya. Protocols for HIV testing and treatment followed 2006 WHO and Ministry of Health (MOH) guidelines. By December 2008, around 2,000 patients were started on treatment at the district level and 1,500 at the rural level in primary health clinics.

From April to September 2008 a cross-sectional survey was conducted. Adults (>18 years old) currently receiving a triple antiretroviral (ARV) drug regimen classified as standard 1^st^ line therapy (e.g. stavudine [d4T] or zidovudine [AZT], lamivudine [3TC] and either nevirapine [NVP] or efavirenz [EFV]) for ≥12 months; ARV-naïve at treatment start; who attended the clinic at least once within the previous 6 months; and given informed consent to participate in the study, were considered eligible for the study.

**Figure 1:**
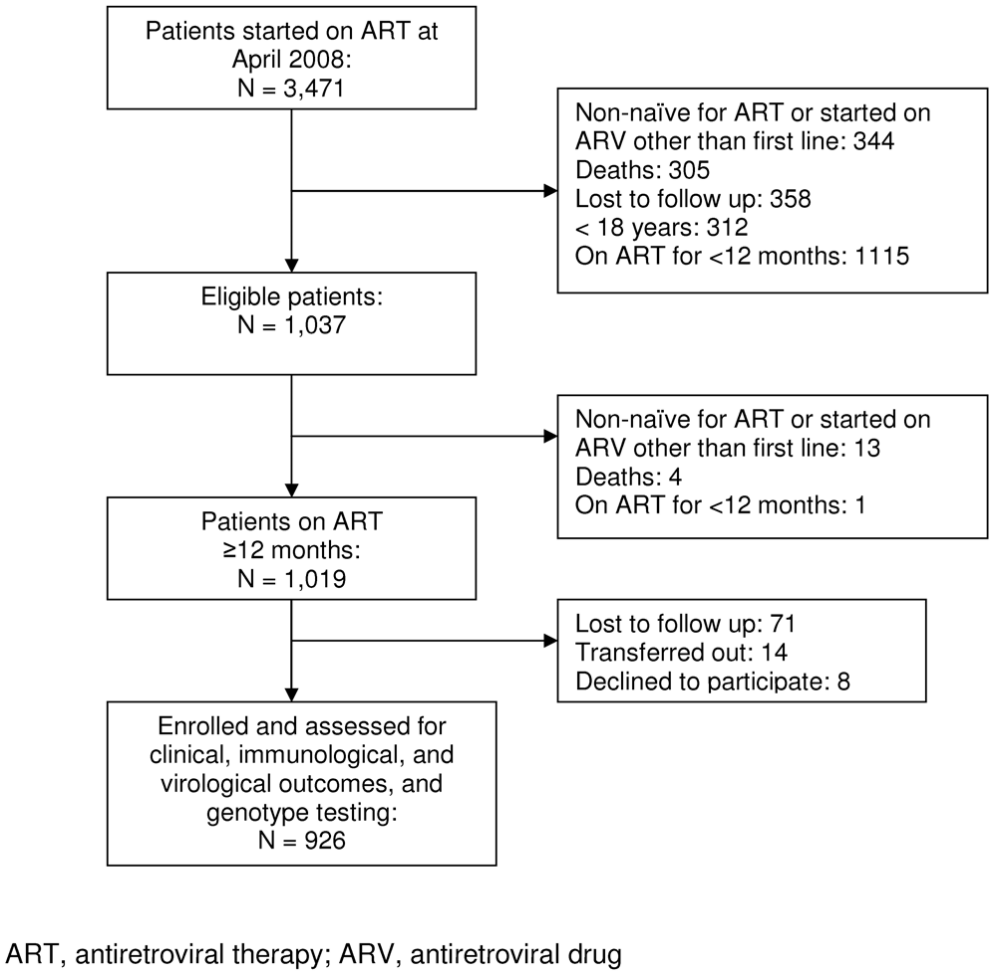
Patient cohort study profile.

All patients meeting the inclusion criteria were placed on a list that was distributed to the clinicians. In addition, a note was added to the front of the medical file for each included patients. If an included patient was attending for a routine visit, the risks and benefits of the study were explained to the patient.

Clinical outcomes were determined based on data routinely recorded in the patients files using data collection software called FUCHIA (Follow Up and Care of HIV Infection and AIDS, Epicentre, Paris France). The data included hospitalization during ART and the occurrence or recurrence of selected WHO stage 3 or 4 conditions during ARV therapy diagnosed by clinical officers trained in HIV care including: weight loss of >10%, pulmonary or extra pulmonary tuberculosis, cryptococcal meningitis, toxoplasmosis, chronic herpes simplex infection, Kaposi’s sarcoma (KS), pneumocystic pneumonia (PCP), HIV wasting syndrome, severe bacterial infections, recurrent severe bacterial pneumonia, lymphoma, persistent oral candidiasis, sepsis/septicaemia, or HIV encephalopathy.

Diagnostic capacities were limited at Busia Hospital, X-ray and acid-fast bacilli in sputum smear were available for TB diagnosis; but specimen culture was unavailable. Lumbar puncture and cerebrospinal fluid latex test (Crypto-LA, Wampole Laboratories, Cranberry, NJ) was used for the diagnosis of cryptococcal meningitis. All other opportunistic infections were diagnosed based on clinical findings.

**Table 1:** Baseline cohort characteristics.

Variable	Number (%)	Median	IQR
	(N = 926)		
**Demographics**			
Gender			
Female	623 (67.3)	–	–
Male	303 (32.7)		
Age at start of ART (years)			
14–19	6 (0.6)	38.3	32.1–44.6
20–29	135 (14.6)		
30–39	378 (40.8)		
40–49	299 (32.3)		
50–59	85 (9.2)		
>60	23 (2.5)		
**Clinical/Immunological/Virological**			
BMI at start of ART (kg/m^2^)			
<16	36 (3.9)	20	18.5–22.0
16–18.5	219 (23.7)		
>18.5	671 (72.5)		
WHO stage at start of ART (n = 923)			
Stage I	43 (4.7)	–	–
Stage II	156 (16.9)		
Stage III	548 (59.4)		
Stage IV	176 (19.1)		
CD4 cell count at start of ART(cells/µL) (n = 919)			
<100	335 (36.5)	133	68–193
100–199	397 (43.2)		
200–299	148 (16.1)		
300–399	37 (4.0)		
≥400	2 (0.2)		
**Treatment**			
Initial ART combination			
d4T 3TC NVP	892 (96.3)	–	–
d4T 3TC EFV	32 (3.5)		
AZT 3TC NVP	2 (0.2)		
AZT 3TC EFV	0 (0.0)		
Median time on treatment (months) (n = 926)			
Duration on ART	–	38	33.8–45.0

IQR, interquartile range; ART, antiretroviral therapy; BMI, body mass index, ARV, antiretroviral drug.

### Laboratory Procedures

On the day of enrollment, a venous blood sample of 10 mL was taken from each patient and divided into two parts: one tested for CD4 cell count at the Busia laboratory using FACSCount flow cytometry (BD, Franklin Lakes, NJ, USA), and the other sent to Kenya Medical Research Institute (KEMRI) in Nairobi, Kenya, where VL testing was performed using NucliSENS EasyQ HIV version 1.2 (bioMérieux, Marcy l’Etoile, France), with limit of detection of 50 copies/mL.

**Table 2:** Viral load results of cross-sectional virological survey.

Viral load, copies/mL	# patients (%), N924
<50*	650 (70.3%)
50–399	126 (13.6%)
400–1,000	49 (5.3%)
1,000–5,000	44 (4.8%)
>5,000	55 (6.0%)

* Limit of detection.

### Definition of Treatment Failure

The treatment protocol of the Busia program recommended measuring CD4 cell count every 6 months and VL in patients with either clinical or immunological failure any time after 12 months on ART. Definitions for treatment failure followed 2006 WHO guidelines. Immunological failure was confirmed with a second CD4 measurement.

**Table 3:** Characteristics of patients with and without virological failure at the time of the study.

Patients without virological failure (<5.000 copies/ml)				Patients with virological failure (>5.000 copies/ml)					
Variable	Number (%)	Median	IQR	Variable	Number (%)	Median	IQR	p value	OR (CI)
	(N = 869)				(N = 55)				
Demographics				Demographics					
Gender				Gender					
Female	587 (67.5)	–	–	Female	34 (61.8)	–	–	0.38	0.78 (0.44–1.36)p = 0.38
Male	282 (32.5)			Male	21 (38.2)				
Age at start of ART (years)				Age at start of ART (years)					
14–19	3 (0.3)	38.5	32.1–44.6	14–19	3 (5.5)	32.4	28.2–44.8	<0.01	1.76 (1.28–2.42)p<0.01
20–29	120 (13.8)			20–29	15 (27.3)				
30–39	357 (41.1)			30–39	21 (38.2)				
40–49	284 (32.7)			40–49	13 (23.6)				
50–59	83 (9.6)			50–59	2 (3.6)				
>60	22 (2.5)			>60	1 (1.8)				
**Clinical/Immunological/Virological**				**Clinical/Immunological/Virological**					
Weight at start of ART (kg)				Weight at start of ART (kg)					
<40	28 (3.2)	55	49–61	<40	3 (5.5)	58	49–64	0.1	0.98 (0.95–1.00)p = 0.1
40–59	567 (65.2)			40–59	28 (50.9)				
60–79	257 (29.6)			60–79	21 (38.2)				
80–99	16 (1.8)			80–99	3 (5.5)				
>100	1 (0.1)			>100	0 (0)				
BMI at start of ART (kg/m^2^)				BMI at start of ART (kg/m^2^)					
<16	35 (4.0)	20	18.5–22.0	<16	1 (1.8)	20.5	18.5–22.5	0.5	0.98 (0.89–1.07)p = 0.67
16–18.5	204 (23.5)			16–18.5	15 (27.3)				
>18.5	630 (72.5)			>18.5	39 (70.9)				
WHO stage at start of ART (n = 866)				WHO stage at start of ART (n = 55)					
Stage I	38 (4.1)	–	–	Stage I	4 (7.3)	–	–		
Stage II	150 (17.0)			Stage II	6 (10.9)				
Stage III	516 (59.1)			Stage III	31 (56.4)				
Stage IV	162 (18.3)			Stage IV	14 (25.5)				
CD4 cell count at start of ART(cells/µL) (n = 55)				CD4 cell count at start of ART(cells/µL) (n = 862)					
<100	309 (35.8)	134	68.7–193	<100	23 (41.8)	116	54–189	0.2	1.00 (0.99–1.01)p = 0.27
100–199	370 (42.9)			100–199	21 (38.2)				
200–299	146 (16.9)			200–299	9 (16.4)				
300–399	35 (4.1)			300–399	2 (3.6)				
≥400	2 (0.2)			≥400	0 (0.0)				

For the study, treatment failure based on CD4 (immunological) criteria was defined as either CD4 count below the patient’s baseline measurement at 6 months of therapy, CD4 count less than 50% of peak measurement at any time after 6 months of therapy, or CD4<100 cells/µL after 12 months of therapy [9].

Treatment failure based on clinical criteria was defined as the occurrence of either new or recurrent disease of WHO clinical stage 3 or 4 at least 6 months after 1^st^ line treatment initiation [9]. Clinical events were not considered for defining treatment failure if they occurred in the first 6 months after ART, as defined by WHO. All clinical events were registered in patient’s files and entered into the database after each consultation.

**Figure 2:**
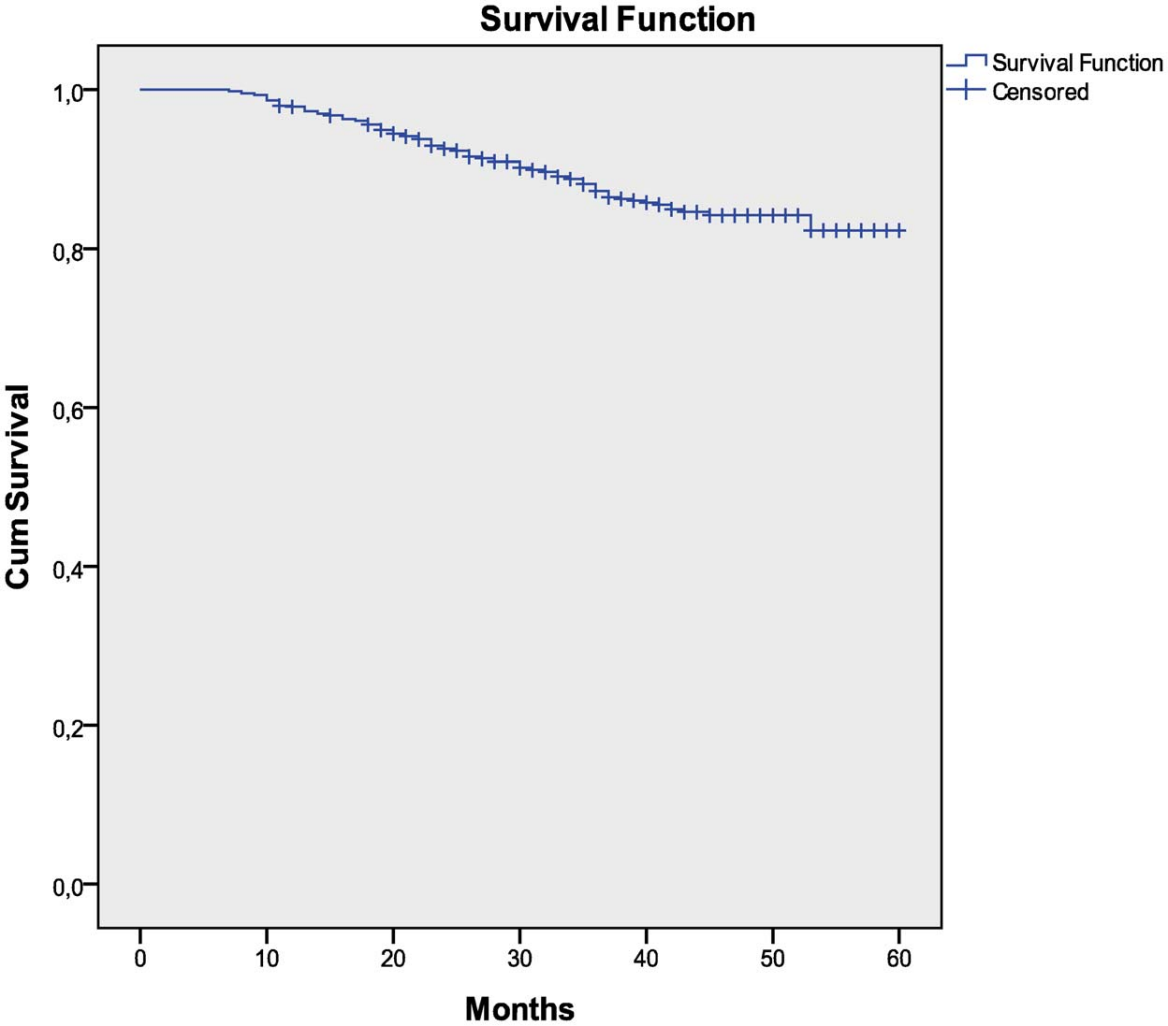
Kaplan Meier analysis from six months of therapy to clinical failure.

**Figure 3:**
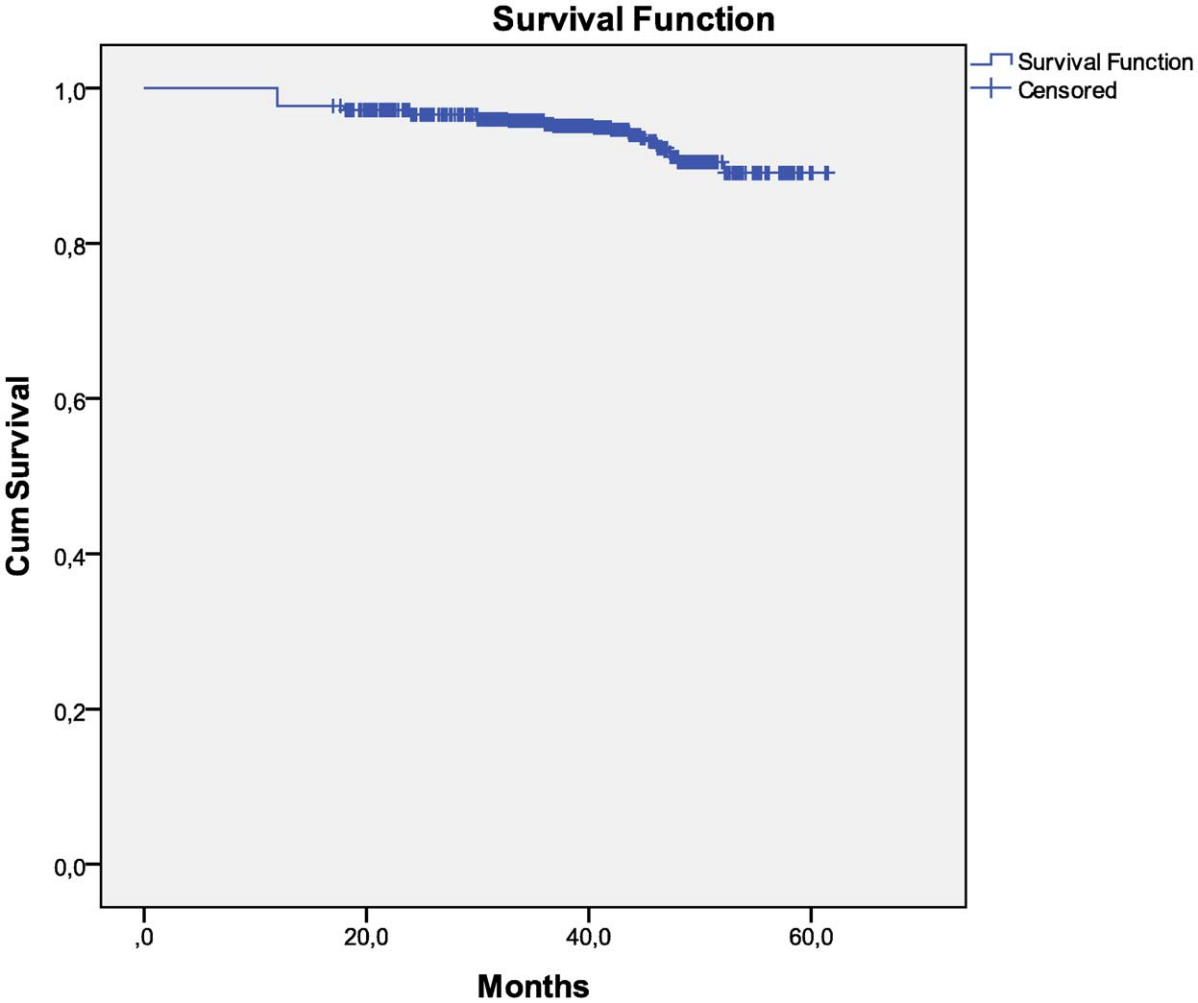
Kaplan Meier analysis from six months of therapy to immunological failure.

Virological failure was assessed by measuring viral load (VL). WHO defines virological failure as plasma HIV-1 RNA level >5,000 copies/mL after 6 months of treatment, if adherence is considered adequate [8], [9].

### Statistical Analysis

Data were entered in FUCHIA software (Epicentre, Paris, France) and exported to and analyzed with SPSS version 18 (Chicago, IL, USA). Data variables were tabulated and analyzed using chi-square and Fisher’s exact tests. *P* values of <0.05 were considered statistically significant.

### Ethical Approval

This study was approved by the Ethics Review Boards of MSF and KEMRI. Only patients who gave informed consent to participate in the study were included in this analysis.

**Table 4:** Sensitivity, specificity, and predictive values of immunological and clinical criteria in identifying virological treatment failure.

Test	TP	FN	FP	TN	Sensitivity,% (95% CI)	Specificity,% (95% CI)	Positive predictivevalue, % (95% CI)	Negative predictivevalue, % (95% CI)
CD4-based immunological failure	13	42	40	829	23.6 (14.4–36.3)	95.4 (93.8–96.6)	24.5 (14.9–37.6)	95.2 (93.5–96.4)
WHO-defined clinical failure	10	45	113	756	18.2 (10.2–30.3)	87.0 (84.6–89.1)	8.1 (4.5–14.3)	94.4 (92.6–95.8)
Combination of both CD4-basedand WHO-defined clinical failure	20	35	143	726	36.4 (24.9–49.6)	83.5 (80.9–85.8)	12.3 (8.1–18.2)	95.4 (93.7–96.7)

TP = true positive, FN = false negative, FP = false positive, TN = true negative.

Results are based in one CD4 and viral load result.

## Results

### Study Population

As of the middle of April 2008, 1,037 out of the 3,471 patients on ART in the MSF Busia HIV/AIDS treatment program were deemed eligible for the study. Those excluded were 1,115 patients on ART <12 months, 344 non-naïve for ART or started on ART other than first line, 305 who died, 358 lost to follow up and 312 who were <18 years old.

After a second analysis of the eligible group, 111 patients were excluded; 13 patients were non-naïve or started on ART other than first line, 71 were lost to follow-up, 14 transferred out during the study, 4 died, 8 declined to participate, and one was on ART <12 months, resulting in 926 patients for the analysis. (Figure 1).

Of the patients in the study, 623 (67.3%) were female. At the start of ART, median age was 38.3 years (interquartile range [IQR] 32.1–44.6); 724 (76.3%) patients were WHO clinical disease stage 3 or 4; the median CD4 cell count was 133 cell/µL [IQR 68–193]; and 255 (27.5%) patients had a body mass index (BMI) <18.5 kg/m^2^ (Table 1).

At the time of study, median CD4 count was 489.4 cells/µL, the median CD4 gain from baseline was 346.4 cells/µL, and median time on ART was 38 months [IQR 33.8–45.0].

ART regimens administered at time of inclusion were d4T/3TC/NVP in 892 (96.3%) patients, d4T/3TC/EFV in 32 (3.5%), and AZT/3TC/NVP in 2 (0.2%), all in standard dosage and qualified fixed-dose combination as per WHO recommendations.

### Treatment Failure

At the time of the study, 123 (13.3%) of 926 patients had clinically defined treatment failure, 53 (5.7%) experienced treatment failure based on CD4 cell count criteria, and 55 (6.0%) had virological failure (VL >5,000 copies/mL) per 2010 WHO definition (Table 2).

Of the patients with clinical failure, 49 (39.8%) had weight loss of >10%, and 73 (59%) had tuberculosis. Opportunistic infections were common; bacterial pneumonia, was reported in 16 (13%) patients, while cryptococcal meningitis and brain toxoplasmosis in 1.6% of the patients.

At time of study, 274/924 (29.7%) of the cohort had detectable VL (detection limit, 50 copies/mL); 650 (70.3%) had <50 copies/ml and 776 (83.9%) did have <400 copies/ml. Of the patients with detectable VL, 46% (126/274) had between 50–399 copies/mL (table 2). Of the 55 patients with VL >5,000 copies/mL, 21 (38.1%) were male; median time on ART was 40 months; 45 (81.8%) were in WHO stage 3 or 4 at the time of ARV start; and median CD4 count at ART start was 124.6 cells/µL. (Table 3).

Comparison of means and proportions were carried out using Mann-Whitney U test and chi square test respectively. The patients who developed a virological failure were statistically younger than the ones who did not develop it (p<0.01). Year of ART initiation was significantly associated with a reduced risk of developing a clinical failure in univariate analysis and it is remained significant also after adjustment for possible confounders in multivariate analysis (p<0.01).

For 773 and 873 patients has been possible to draw a Kaplan Meier analysis respectively from six months of therapy to clinical and immunological failure. The mean time before developing clinical failure is 54.5 months (SD 0.48, CI 95%) and for immunological failure is 58.6 months (SD 0.39, CI 95%). (Figure 2, 3).

### Sensitivity, Specificity, and Positive and Negative Predictive Values

Sensitivity of immunological and clinical WHO criteria to define treatment failure was 23.6% and 18.2%, respectively, compared with virological failure (Table 4). When combining immunological and clinical failure (patient having either one or both) and comparing with virological outcome, sensitivity was 36.4% for predicting virological failure.

Specificities for immunological, clinical, and both together for predicting virological failure were 95.4%, 87.0%, and 83.5%, respectively.

Positive predictive values (PPV) for immunological and clinical criteria to define virological failure were 24.5% and 8.1%, respectively. When both criteria were analyzed together, PPV was 12.3%. Negative predictive values (NPV) were 95.2%, 94.4%, and 95.4%, respectively, for immunological, clinical, and both criteria.

## Discussion

In our study we found a relatively low proportion of virological failure (6.0%) following 2010 WHO definition (VL >5000 copies/ml) in patients on ART for more than 12 months as reported in other LMICs, supporting the fact that ART can be provided in resource-poor settings with favorable outcomes [7], [11], [12].

In this study, the PPV of clinical or immunological monitoring for detecting virological treatment failure was relatively low. Mee et al reported a PPV of CD4 count of 36.8% while Kaiser et al recorded PPV ranging from 9.5%–28.7%. [11], [13], [14], [15], [16], [17], which could result in patients with adequate viral suppression being incorrectly identified as failing treatment and being unnecessarily switched to second-line therapy [18]. This would not only reduce treatment options for patients but also potentially increase costs and make follow-up of patients receiving protease inhibitors more difficult. An algorithm for determining treatment failure based on clinical history, hemoglobin level, and CD4 cell count has recently been proposed, but it has not been validated in routine clinical care [19].

Low sensitivity of clinical and immunological criteria to define treatment failure highlights the need for improved methods to detect treatment failure in the absence of VL testing. In our study, only 8.1% of patients with clinical failure and 24.5% of those with immunological failure were found to have virological failure.

Only 3/55 (5.45%) of patients with confirmed virological failure met both clinical and immunological criteria for treatment failure, and 35/55 (63%) of the patients with virological failure did not meet both clinical and immunological definitions of failure, showing that patients with VL >5,000 copies/mL might not meet any of the currently used criteria to detect treatment failure. Many treatment failures may therefore be missed using only clinical and immunological criteria, which could lead to accumulated resistance in patients who continue on failing regimens.

An evaluation from resources limited countries found no evidence of improved mortality in programs with viral load test, though follow-up was short. [20]. On the other hand, several studies have concluded that clinical indicators and CD4 cell count are not favorable predictors of virological failure and routine laboratory monitoring is associated with improved health and survival when compared with clinical monitoring alone. [14], [17], [20] In HIV high-prevalence, resource-poor settings, where task shifting takes place to scale up ART, sensitive models are needed to accurately detect treatment failure when VL testing is unavailable.

These results from a Kenyan ART program also illustrate the difficulties faced by other African countries in implementing the new WHO recommendations for ART initiation [8], moving to improved first-line regimens containing tenofovir (TDF) or zidovudine (AZT) in patients who have already been treated with d4T-based regimen and might have treatment failure. In the ideal scenario of universal access to VL testing, every patient could be assessed before being switched from a first- to second-line regimen, but because this is not the case in most resource-limited settings, many patients might be switched to a regimen which is the only available second-line therapy in LMICs.

Follow-up of patients with VL measurements seems to be the only way to adequately monitor the patients, and VL appears to be the most reliable tool for deciding when to switch failing regimens for patients [14], [17]. In programs with access to VL monitoring, patients tended to switch treatment earlier and at higher CD4 cell counts than at sites without VL [21]. Despite the evidence, VL testing is not yet widely available for monitoring of patients on ART in resource-poor settings, and no other simple tools exist for treatment failure detection. New VL assays meeting specifications for use in resource-constrained settings are urgently needed to tackle the current needs of ART monitoring and clinical assistance for treatment decision-making.

A strength of our study was in the assessment of clinical outcomes since they were systematically collected in patient’s files, allowing us to examine the correlation between clinical and immunological criteria together with viral load measurement. Another strength of the study was that it was done in a routine ART program in a resource-limited setting including decentralized rural clinics, which reflects the reality of other sub-Saharan African countries.

A limitation of the study lay in not being able to analyze adherence despite the data obtained through questionnaires since it was impossible to find standard definitions using the current self-reported and visual-analogue scale. Another study weakness was the limited diagnostic capacities for the main opportunistic infections seen in our program, which could bias some of the clinical events registered.

This study builds on existing literature and builds the case that clinical and immunologic criteria, given low sensitivity, allow for individuals to switch to expensive second-line who may not have true virological failure.

In conclusion, these data illustrate the urgent needs for new or improved algorithms for measuring clinical or immunological treatment failure and wider access to VL monitoring in low-resource settings. Using current WHO immunological and clinical criteria to determine virological treatment failure is inadequate in a setting were VL is not widely available and second-line ART options are limited.

## References

[1] WHO: HIV/AIDS program highlights 2008–2009. Available: http://www.who.int/hiv/pub/9789241599450/en/index.html. Accessed 18 October 2012.

[2] WHO/UNAIDS/UNICEF: Towards universal access: scaling up priority HIV/AIDS interventions in the health sector. Progress report June 2008. Available: http://www.who.int/hiv/pub/2008progressreport/en/. Accessed 18 October 2012.

[3] UNAIDS: Report on the global AIDS epidemic 2010. Available: http://www.unaids.org/globalreport/global_report.htm. Accessed 18 October 2012.

[4] Braitstein P, Brinkhof MW, Dabis F, Schechter M, Boulle A, et al (2006) Mortality of HIV-1-infected patients in the first year of antiretroviral therapy: comparison between low-income and high-income countries. Lancet 367: 817–824. 16530575 10.1016/S0140-6736(06)68337-2

[5] Rosen S, Fox MP, Gill CJ (2007) Patient retention in antiretroviral therapy programs in sub-Saharan Africa: a systematic review. PLoS Med 4: e298. 17941716 10.1371/journal.pmed.0040298 PMC2020494

[6] Elliott JH, Lynen L, Calmy A, De Luca A, Shafer RW, et al (2008) Rational use of antiretroviral therapy in low-income and middle-income countries: optimizing regimen sequencing and switching. AIDS 22: 2053–2067. 18753937 10.1097/QAD.0b013e328309520d

[7] Tassie JM, Szumilin E, Calmy A, Goemaere E (2003) Highly active antiretroviral therapy in resource-poor settings: the experience of Médecins Sans Frontières. AIDS 17: 1995–1997. 12960837 10.1097/00002030-200309050-00023

[8] WHO: Rapid advice: antiretroviral therapy for HIV infection in adults and adolescents, November 2009. Available: http://www.who.int/hiv/pub/arv/advice/en/index.html. Accessed 18 October 2012.

[9] WHO: Antiretroviral therapy for HIV infection in adults and adolescents: Recommendations for a public health approach, 2010 revision. Available: http://whqlibdoc.who.int/publications/2010/9789241599764_eng.pdf Accessed 18 October 2012. 23741771

[10] WHO: WHO consultation on technical and operational recommendations for scale-up of laboratory services and monitoring HIV antiretroviral therapy in resource-limited settings, 2005. Available: http://www.who.int/hiv/pub/meetingreports/scaleup/en/index.html Accessed 18 October 2012.

[11] Barth R, Schim van der Loeff M, Schuurman R, Hoepelman A, Wensing A M J (2010) Virological follow-up of adult patients in antiretroviral treatment programs in sub-Saharan Africa: a systematic review. Lancet Infect Dis 10: 155–66. 20185094 10.1016/S1473-3099(09)70328-7

[12] Mermin J, Ekwaru J, Were W, Degerman R, Bunnell R, et al (2011) Utility of routine viral load, CD4 cell count, and clinical monitoring among adults with HIV receiving antiretroviral therapy in Uganda. BMJ 343: d6792 doi: 10.1136/bmj.d6792 22074711 10.1136/bmj.d6792 PMC3213241

[13] Mee P, Fielding KL, Charalambous S, Churchyard GJ, Grant AD (2008) Evaluation of the WHO criteria for antiretroviral treatment failure among adults in South Africa. AIDS 22: 1971–1977. 18784460 10.1097/QAD.0b013e32830e4cd8

[14] Calmy A, Ford N, Hirschel B, Reynolds SJ, Lynen L, et al (2007) HIV viral load monitoring in resource-limited regions: optional or necessary? Clin Infect Dis 44: 128–134. 17143828 10.1086/510073

[15] Bagchi S, Kempf MC, Westfall AO, Maherya A, Willig J, et al (2007) Can routine clinical markers be used longitudinally to monitor antiretroviral therapy success in resource-limited settings? Clin Infect Dis 44: 135–8. 17143829 10.1086/510072

[16] Keiser O, Mac Phail P, Boulle A, Wood R, Schechter M, et al (2009) Accuracy of WHO CD4 cell count criteria for virological failure of antiretroviral therapy. Trop Med and Intl Health 14: 1220–1225. 10.1111/j.1365-3156.2009.02338.x PMC3722497 19624478

[17] Reynolds S, Nakigozi G, Newell K, et al (2009) Failure of immunologic criteria to appropriately identify antiretroviral treatment failure in Uganda. AIDS 23(6): 697–700. 19209067 10.1097/QAD.0b013e3283262a78 PMC2720562

[18] Moore DM, Mermin J, Awor A, Yip B, Hogg RS, et al (2006) Performance of immunologic responses in predicting viral load suppression: implications for monitoring patients in resource-limited settings. J Aquir Immune Defic Syndr 43: 436–9. 10.1097/01.qai.0000243105.80393.42 17019367

[19] Colebunders R, Moses KR, Laurence J, Shihab HM, Semitala F, et al (2006) A new model to monitor the virological efficacy of antiretroviral treatment in resource poor countries. Lancet Infect Dis 6: 53–59. 16377535 10.1016/S1473-3099(05)70327-3

[20] Braitstein P, Brinkhof MW, Dabis F, Schechter M, Boulle A, et al (2006) Mortality of HIV-1-infected patients in the first year of antiretroviral therapy: comparison between low-income and high-income countries. Lancet 367(9513): 817–824. 16530575 10.1016/S0140-6736(06)68337-2

[21] Keiser O, Tweya H, Boulle A, Braitstein P, Schecter M, et al (2009) Switching to second-line antiretroviral therapy in resource-limited settings: comparison of programs with and without viral load monitoring. AIDS 23: 1867–1874. 19531928 10.1097/QAD.0b013e32832e05b2 PMC2956749

